# Apoptosis Inhibitor of Macrophage and Acute Inflammation in IgA Nephropathy

**DOI:** 10.34067/KID.0000001202

**Published:** 2026-05-22

**Authors:** Rina Kato, Hitoshi Suzuki, Ryosuke Aoki, Ayako Koizumi, Yusuke Fukao, Maiko Nakayama, Yoshihito Nihei, Toshiki Kano, Yuko Makita, Masahiro Muto, Keisuke Yasuda, Satoko Arai, Toru Miyazaki, Yusuke Suzuki

**Affiliations:** 1 Department of Nephrology, Juntendo University Faculty of Medicine, Tokyo, Japan; 2 The Institute for AIM Medicine, Tokyo, Japan; 3 Laboratory of Food Chemistry, Department of Applied Biological Chemistry, Graduate School of Agricultural and Life Sciences, The University of Tokyo, Tokyo, Japan

**Keywords:** complement, IgA nephropathy

## Abstract

**Key Points:**

- Serum IgM-free apoptosis inhibitor of macrophage reflects disease activity in human IgA nephropathy.
- Glomerular apoptosis inhibitor of macrophage deposition was colocalized with mesangial IgA and reflected the acute glomerular injuries by complement activation.
- Multivariate Cox regression analysis revealed serum IgM-free apoptosis inhibitor of macrophage as an independent predictor of hematuria remission in patients.

**Background:**

IgA nephropathy is characterized by mesangial deposits of IgA1-containing immune complex with complement 3. Our previous study demonstrated that apoptosis inhibitor of macrophage (AIM) plays a critical role in *in situ* IgA-containing immune complex formation and subsequent complement activation in murine IgA nephropathy. We further evaluated the clinicopathologic role of AIM in human IgA nephropathy.

**Methods:**

We enrolled 77 patients with IgA nephropathy, five patients with thin basement membrane disease as disease controls, and 40 age-matched healthy individuals as healthy controls. We analyzed the association between serum IgM-free AIM (sAIM)/glomerular AIM (gAIM) deposition area and clinicopathologic features.

**Results:**

sAIM level was higher in patients with IgA nephropathy at diagnosis than in disease and healthy controls (1.02±0.49 versus 0.56±0.19 versus 0.57±0.20 *µ*g/ml), significantly decreased to 0.67±0.44 *µ*g/ml after tonsillectomy and steroid pulse therapy, and was associated with serum IgA (*r*^2^=0.23) and IgA–IgG immune complex levels (*r*^2^=0.12). gAIM was colocalized with mesangial IgA and high intensity of gAIM was associated with the degree of hematuria (30–49 versus 10–19 per high-power field) and acute lesions, including those with Oxford classification scores of endocapillary hypercellularity (E1 3% versus E0 2%) and crescent formation (C1 3% versus C0 2%). Furthermore, gAIM deposition was positively correlated with glomerular complement 3 deposition (*r*^2^=0.54). Multivariate Cox regression analysis revealed sAIM as an independent predictor of hematuria remission (hazard ratio, 1.86; 95% confidence interval, 1.03 to 3.15). According to Kaplan–Meier analysis, patients with larger reduction of sAIM (≥0.45 *µ*g/ml) after tonsillectomy and steroid pulse therapy showed a significantly improved hematuria (6 versus 9 months).

**Conclusions:**

We suggest that IgA nephropathy patients with high sAIM levels have high disease activity and patients with high intensity of gAIM deposition have acute glomerular injuries leading to hematuria.

## Visual Abstract

**Figure:**
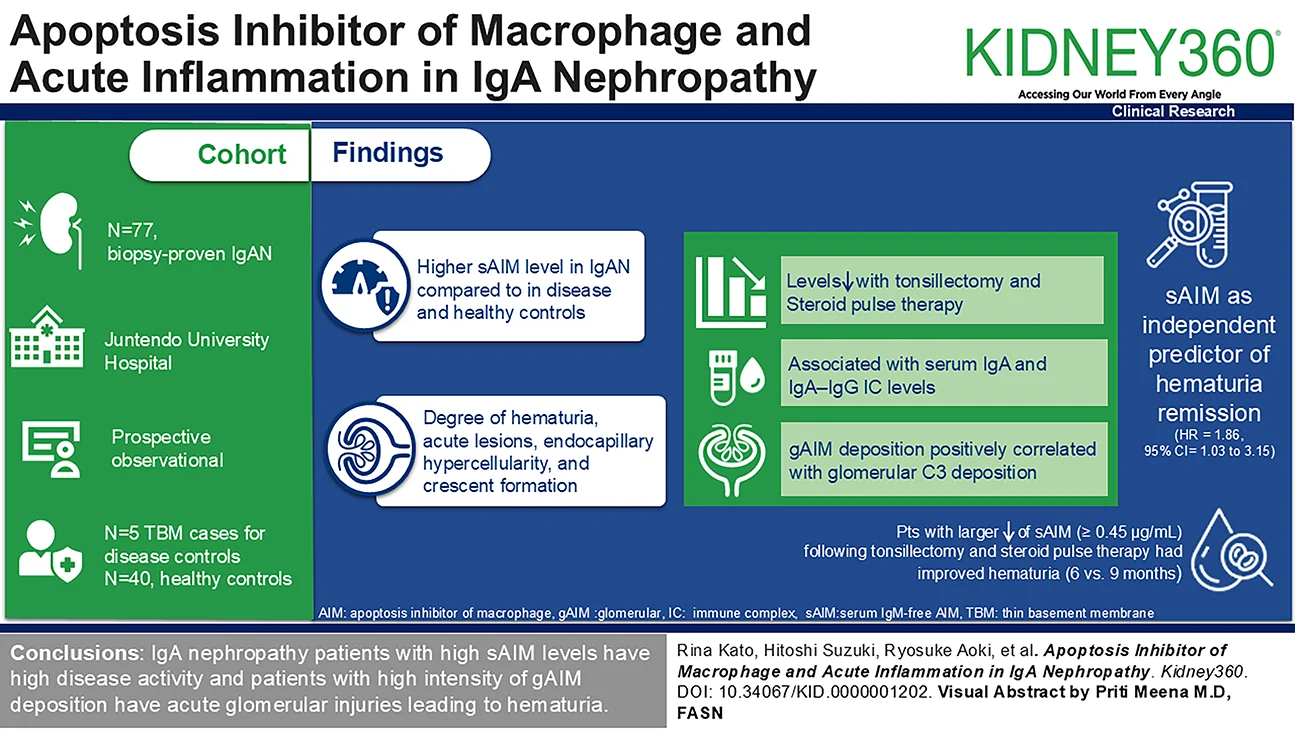

## Introduction

IgA nephropathy is the most common primary GN worldwide and a frequent cause of ESKD.^1^ Microscopic hematuria accompanied by variable amounts of proteinuria is the most common clinical manifestation of IgA nephropathy, which is characterized by predominantly mesangial IgA and variable IgG and IgM deposition.^1^ The widely accepted pathogenesis of IgA nephropathy has been the multihit hypothesis, which involves the production of galactose-deficient IgA1 (Gd-IgA1, hit 1), IgG or IgA autoantibodies that recognize Gd-IgA1 (hit 2), and subsequent immune complex formation (hit 3) and glomerular deposition that activates mesangial cells and incites glomerular injuries (hit 4).^2–4^

Several studies have indicated that immune complex formation including autoantibodies of Gd-IgA1 plays a critical role in IgA nephropathy progression. Both *in vitro* and *in vivo* studies have demonstrated that immune complex containing Gd-IgA1 and Gd-IgA1‑specific IgG autoantibodies, not IgA alone, are necessary to induce kidney injury.^5,6^ In fact, serum level of immune complex containing Gd-IgA1 correlated with the degree of hematuria, and codeposition of glomerular IgM and IgG correlated with histologic severity in human IgA nephropathy.^7–9^ Although routine immunofluorescence microscopy failed to show IgG in up to 50%–80% of kidney biopsies, the presence of Gd-IgA1‑specific IgG autoantibodies has been reported in kidney biopsies of all patients with IgA nephropathy.^10^ Therefore, Gd-IgA1–IgG immune complex formation is believed to have an important effect on IgA nephropathy progression.

Apoptosis inhibitor of macrophage (AIM) or CD5L, which is encoded by the *Cd5l* gene, was reported to play a key role in immune complex formation and kidney injury in IgA nephropathy.^11^ AIM is produced by resident tissue macrophages and was initially found to support the survival of macrophages exposed to apoptosis-inducing stimuli.^12^ In circulation, AIM binds with an IgM pentamer, which protects it from renal excretion and maintains high levels of circulating AIM (IgM-bound AIM).^13,14^ Interestingly, during AKI, AIM is dissociated from IgM (IgM-free AIM) and accumulates on obstructed renal proximal tubules, contributing to the phagocytic removal of dead cell debris.^15^ This dead cell debris–clearing function of AIM has also been implicated in promoting resolution of inflammation in fungus-induced peritonitis and in suppressing ocular hypertension.^16,17^ In addition, IgM-free AIM binds to various damage-associated molecular patterns, facilitating their phagocytic clearance by macrophages and thereby promoting tissue repair in ischemic stroke.^14,18^

In a previous study, we found that glomerular AIM (gAIM) was codeposited with IgA in patients with IgA nephropathy and in an IgA nephropathy-prone grouped Deutschland, Denken, and Yoken (gddY) mouse model.^19^ Remarkably, the AIM-deficient (AIM−/−) gddY mice generated by the crisperclustered regularly interspaced short palindromic repeats/Cas9 system showed large absence of abnormal urinary findings and glomerular deposition of IgM, IgG, and complement 3 (C3).^11^ In addition, administration of recombinant AIM to AIM−/− gddY mice restored glomerular IgA/IgM/IgG codeposition and complement activation involving intense glomerular injury.^11^ On the basis of persistent glomerular IgA deposition in AIM−/− gddY mice and the absence of AIM deposition in IgA−/− gddY mice, the involvement of AIM in glomerular inflammation was suggested to be through *in situ* IgM–IgG immune complex formation after IgA deposition.^11^ However, the mechanism of systemic and/or *in situ* AIM in the pathogenesis of human IgA nephropathy remains unclear.

The aim of this study was to further elucidate the clinical significance of AIM by histologic and serologic analyses. We evaluated the association between glomerular or circulating AIM and disease severity or prognosis in patients with IgA nephropathy.

## Methods

### Study Population

A total of 115 patients with renal biopsy-proven IgA nephropathy and five patients with thin basement membrane disease participated in this study (Supplemental Figure 1). The exclusion criteria were (*1*) age younger than 18 years, (*2*) IgA nephropathy in a transplanted kidney, (*3*) previous treatment of IgA nephropathy, (*4*) presence of other comorbid kidney diseases, (*5*) eGFR <60 ml/min per 1.73 m^2^, and (*6*) insufficient samples at diagnosis. Serum samples from 40 age-matched healthy blood donors without indicators of kidney disease were used as healthy controls. This study complied with the Declaration of Helsinki principles and was approved by the Ethics Committee of Juntendo University Hospital (H19-0239, M19-0223). Informed consent was obtained from all enrolled individuals.

### Treatment and Follow-Up

All the treatments were recorded, including steroid pulse, oral corticosteroid, other immunosuppressive drugs, tonsillectomy, and renin-angiotensin system inhibitors. A total of 54 patients underwent tonsillectomy and steroid therapy. Their treatment details and follow-up schedule are shown in Supplemental Figure 2. Steroid pulse therapy (500 mg per day of methylprednisolone intravenously for 3 consecutive days, thrice every 2 months) was administered after tonsillectomy. Between sessions of steroid pulse therapy, patients were prescribed oral prednisolone (0.5 mg/kg ideal body weight) on alternate days. After last steroid pulse therapy, patients went for follow-up visits every month, and the dose of oral prednisolone was gradually tapered off. All serum and urine tests were performed at renal biopsy performance, the beginning of all treatments, and follow-up visits. The magnitude of hematuria was graded as <5, 5–9, 10–19, 30–49, and >50 per high-power field (/HPF). Hematuria remission was defined as three consecutive red blood cell count of <5/HPF for at least 6 months, and proteinuria remission was defined as three consecutive proteinuria of <0.3 g/gCr for at least 6 months.^20^

### Renal Biopsy

All patients were diagnosed on the basis of optical, immunofluorescence, and electron microscopy. Pathologic variables were evaluated using the Oxford classification.^21,22^ In immunofluorescence, glomerular IgA, IgG, IgM, C3, and C1q intensities were graded as 0 (absent), 1+, and 2+.

### Measurement of Serum Levels of AIM, IgA–IgG Immune Complex, IgA–IgM Immune Complex, and Gd-IgA1

Serum IgM-free AIM (sAIM) level was measured by ELISA using mouse anti-human AIM monoclonal antibodies (clones 11 and 12).^23^ The cutoff value of sAIM level at biopsy was defined as mean+1.96 SD of healthy controls. Using the cutoff value, all patients were divided into the high (≧0.97 *µ*g/ml) and low (<0.97 *µ*g/ml) sAIM group. All patients with tonsillectomy and steroid pulse therapy measured sAIM levels at diagnosis and on the last day of steroid pulse therapy, and sAIM reduction during the therapy (ΔsAIM) was calculated. ELISA was used to measure serum IgA–IgG immune complex levels, according to our previous protocol,^7^ and IgA–IgM immune complex. High-adsorption polystyrene 96-microwell plates were coated with 1 *µ*g/ml of goat anti-human IgA (Bethyl Laboratories, Montgomery, TX). After washing and blocking with 1% BSA in PBS, samples were diluted 100-fold with the same buffer. The captured Ig was detected using horseradish peroxidase-labeled goat IgG anti-human IgM (Invitrogen, Carlsbad, CA). Color reaction was stopped with 1-M sulfuric acid, and absorbance was measured at 490 nm using the EL312 BioKinetiC Microplate Reader (BioTek, Winooski, VT). The serum levels of Gd-IgA1 were measured by ELISA using KM55 mAb, according to the manufacturer's protocol (Immuno-Biological Laboratories, Fujioka, Japan).^3^

### Immunofluorescence Staining and Histologic Analysis

Immunofluorescence staining of frozen tissue samples was performed to determine colocalization of AIM with complement components (C3, factor B, C5b-9, and associated serine protease-2 [MASP-2]). Renal specimens mounted in optimal cutting temperature compound 4583 (Sakura Finetek Japan Co., Ltd., Tokyo, Japan) were sliced into 4-*µ*m sections and stored at −80°C. The sections were immersed in PBS for 15 minutes and then in a blocking solution (0.2% fish gelatin and 2% BSA with PBS).

Subsequently, the sections were incubated overnight at 4°C with the primary rabbit anti-AIM antibody (rab2 rabbit polyclonal for mice and human; customized at our laboratory and fully confirmed for its specificity^11^) at 1:500 dilution in PBS. After washing three times with PBS, Alexa 555‑conjugated antirabbit IgG (A31572; Invitrogen) was added for 30 minutes at room temperature. After washing with PBS three times for 5 minutes, the sections were incubated for 60 minutes at 37°C with rabbit anti-human C3 (F0201; Dako, Santa Clara, CA), rabbit anti-human factor B (NBP1-89985; Novus, Centennial, CO), rabbit anti-human C5b-9 (bs-2673R-A488, Bioss, Boston, MA), and mouse anti-human MASP-2 (sc-390200; Santa Cruz Biotechnology, Dallas, TX). After another wash, an appropriate fluorophore-conjugated secondary antibody (1:300 dilution in PBS) was added to each anti-human factor B and anti-human MASP-2 for 30 minutes at room temperature.

Specimens were analyzed using confocal laser microscopy (FV-1000; Olympus Corporation, Tokyo, Japan). Using the whole glomeruli from each specimen (≥3 per sample), we measured the percentage area of positive staining for AIM and complement components using analyzing software (KS400, Zeiss, Oberkochen, Germany); the average was recorded. On the basis of the median gAIM deposition area, we divided the patients into the high-intensity (≥4.0%) and low-intensity (<4.0%) groups.

### Statistical Analyses

Statistical analysis was performed using GraphPad Prism version 9 (GraphPad Software, San Diego, CA). Normally distributed variables, including sAIM levels, are presented as mean±SD and compared using either *t* test or Pearson correlation coefficients as appropriate. Non-normally distributed variables, including gAIM deposition area, IgA–IgG immune complex, and IgA–IgM immune complex, were expressed as median and interquartile ranges and compared using Mann–Whitney *U* test or Spearman correlation coefficients. Categorical variables were expressed as frequencies and percentages and compared using Fisher and chi-square tests. The sAIM levels were compared before and after treatment using the paired *t* test. Univariate and multivariate Cox proportional hazard models were used to explore the influence of several variables on the occurrence of hematuria remission, estimating hazard ratios (HRs) with 95% confidence intervals (CIs). Cumulative survival curves were derived using the Kaplan–Meier method and compared using log-rank test. *P* values < 0.05 were considered statistically significant.

## Results

### Clinical and Histologic Characteristics of Patients with IgA Nephropathy

The 77 biopsied patients with IgA nephropathy had a mean±SD age of 39±15 years, eGFR of 84±18 ml/min per 1.73 m^2^, and median (interquartile range) urine protein of 0.6 (0.3–1.3) g/gCr. According to the Oxford classification,^21,22^ 25%, 62%, 64%, 22%, and 30% of patients had mesangial hypercellularity (M1), endocapillary hypercellularity (E1), segmental glomerulosclerosis (S1), tubular atrophy/interstitial fibrosis (T1), and crescent (C1), respectively (Table 1).

**Table 1:** Relationship between serum IgM-free apoptosis inhibitor of macrophage levels with clinical characteristics of patients with IgA nephropathy

Variable	Patients With IgA Nephropathy (*n*=77)	High-sAIM Group (≧0.97 *µ*g/ml; *n*=39)	Low-sAIM Group (<0.97 *µ*g/ml; *n*=38)
**Clinical features**			
Sex: male/female, *n*	38/39	22/17	16/22
Age, yr, mean±SD	39±15	37±9	40±19
MAP, mm Hg, mean±SD	83±9	84±10	84±9
BMI, mean±SD	23±4	24±4	23±4
**Laboratory measurements**			
Hematuria (<5, 5–9, 10–19, 30–49, ≧50/HPF), *n* (%)	5 (7), 10 (13), 16(21), 12 (17), 34 (44)	2 (5), 4 (10), 6 (15), 8 (21), 19 (49)	3 (8), 6 (16), 10 (26), 4 (11), 15 (40)
Proteinuria, g/gCr, median (IQR)	0.6 (0.3–1.3)	0.7 (0.3–1.3)	0.5 (0.4–1.3)
Serum creatinine, mg/dl, mean±SD	0.8±0.2	0.8±0.2	0.7±0.2
eGFR, ml/min per 1.73 m^2^, mean±SD	84±18	82±17	85±19
Serum albumin, g/dl, mean±SD	4.0±0.5	4.1±0.5	4.1±0.4
Serum IgG, mg/dl, mean±SD	1041±304	1067±376	1005±221
Serum IgA, mg/dl, mean±SD	307±90	342±97	266±65
Serum IgM, mg/dl, mean±SD	100±47	95±53	99±39
Serum C3, mg/dl, mean±SD	97±18	101±18	93±18
Serum Gd-IgA1, mg/dl, median (IQR)	4.5 (3.6–5.7)	4.7 (4.0–6.6)	4.2 (3.2–5.2)
Urine Gd-IgA1/UTPCr, g/gCr, median (IQR)	29.7 (17.2–66.4)	33.4 (20.9–66.4)	27.3 (13.1–53.1)
Serum IgA–IgG immune complex, OD, median (IQR)	1.71 (1.63–1.83)	1.79 (1.67–1.88)	1.66 (1.59–1.76)
Serum IgA–IgM immune complex, OD, median (IQR)	1.78 (1.42–1.99)	1.68 (1.36–1.91)	1.83 (1.53–2.05)
**Pathologic findings**			
Oxford classification, *n* (%)			
*Mesangial proliferation (M) 1*	19 (25)	13 (33)	6 (16)
*Endocapillary cellularity (E) 1*	48 (62)	26 (67)	22 (58)
*Segmental sclerosis (S) 1*	49 (64)	28 (72)	21 (55)
*Tubular atrophy/Interstitial fibrosis (T) 1*	17 (22)	5 (13)	12 (32)
*Crescent (C) 1*	23 (30)	9 (23)	14 (37)
Immunofluorescence, *n* (%)			
*Glomerular IgA deposition (0, 1+, 2+)*	0 (0), 0 (0), 77 (100)	0 (0), 0 (0), 39 (100)	0 (0), 0 (0), 38 (100)
*Glomerular IgG deposition (0, 1+, 2+)*	22 (29), 55 (71), 0 (0)	13 (33), 26 (67), 0 (0)	9 (24), 29 (76), 0 (0)
*Glomerular IgM deposition (0, 1+, 2+)*	19 (25), 56 (73), 2 (3)	12 (31), 25 (64), 2 (5)	7 (18), 31 (82), 0 (0)
*Glomerular C3 deposition (0, 1+, 2+)*	0 (0), 20 (26), 57 (74)	0 (0), 11 (28), 28 (72)	0.0 (0), 9 (24), 29 (76)
*Glomerular C1q deposition (0, 1+, 2+)*	77 (100), 0 (0), 0 (0)	39 (100), 0 (0), 0 (0)	38 (100), 0 (0), 0 (0)
gAIM deposition, %, median (IQR)	2.7 (1.5–4.4)	2.9 (1.6–4.3)	2.1 (1.3–4.4)
**Treatment options, *n* (%)**			
Tonsillectomy and steroid pulse therapy	54 (70)	28 (76)	25 (63)
Steroid pulse mono therapy	3 (4)	1 (3)	2 (5)
Tonsillectomy only	14 (18)	7 (18)	7 (18)
RAS inhibitors	24 (31)	11 (28)	13 (34)

Significance was determined by the Mann–Whitney *U* test and unpaired *t* test. BMI, body mass index; C3, complement 3; gAIM, glomerular apoptosis inhibitor of macrophage; Gd-IgA1, galactose-deficient IgA1; /HPF, per high-power field; IQR, interquartile range; MAP, mean arterial BP; RAS, renin-angiotensin system; sAIM, serum IgM‑free apoptosis inhibitor of macrophage; UTPCr, urinary total protein-to-creatinine ratio.

### sAIM Level in Patients with IgA Nephropathy

**Figure 1:**
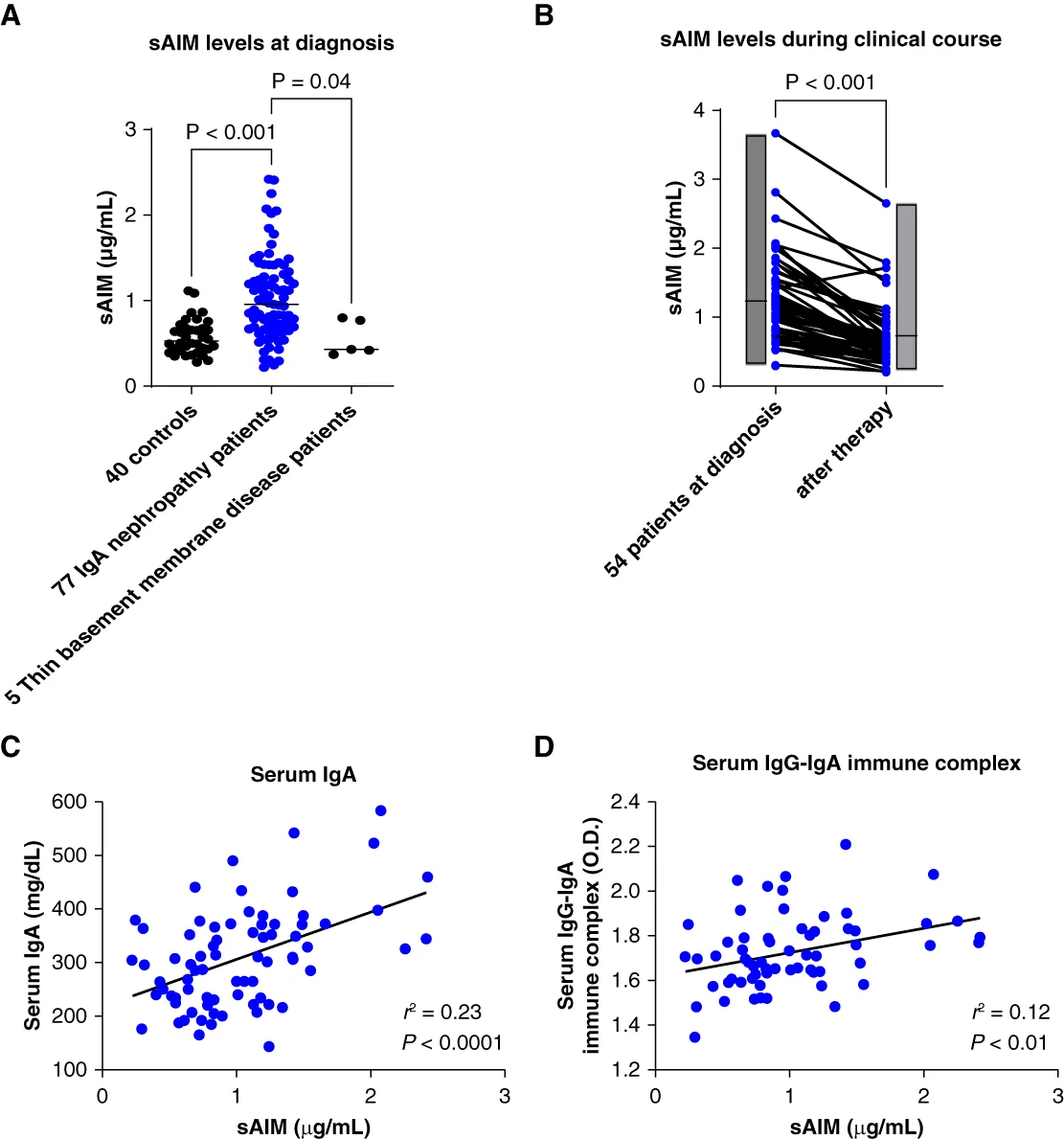
**sAIM level in patients with IgA nephropathy at biopsy and during the clinical course.** (A) The sAIM level is significantly higher in 77 patients with IgA nephropathy than in 40 healthy controls (1.02±0.49 versus 0.57±0.20 *µ*g/ml; *P* < 0.0001) and five patients with thin basement membrane disease (versus 0.56±0.19 *µ*g/ml; *P* < 0.05). (B) Fifty-four patients who underwent tonsillectomy and steroid therapy significantly decreased throughout the therapy (versus 0.67±0.44 *µ*g/ml, *P* < 0.0001). (C) The sAIM level in 77 patients with IgA nephropathy is positively correlated with the serum levels of IgA (*r*^2^=0.23, *P* < 0.0001) and (D) IgA–IgG immune complex (*r*^2^=0.12, *P* < 0.01) at diagnosis. sAIM, serum IgM-free apoptosis inhibitor of macrophage.

We found that the sAIM level was significantly higher in patients with IgA nephropathy than in healthy controls (1.02±0.49 versus 0.57±0.20 *µ*g/ml; *P* < 0.0001; Figure 1A) and patients with thin basement membrane disease (versus 0.56±0.19 *µ*g/ml; *P* = 0.04). sAIM level significantly decreased after tonsillectomy and steroid pulse therapy (versus 0.67±0.44 *µ*g/ml, *P* < 0.0001; Figure 1B). Compared with the low-sAIM group, the high-sAIM group had significantly higher serum levels of IgA (342±97 versus 266±65 mg/dl, *P* < 0.001) and IgA–IgG immune complex (1.79 OD [1.67–1.88 OD] versus 1.66 OD [1.59–1.76 OD], *P* < 0.01; Table 1). Furthermore, sAIM level was positively correlated with the serum levels of IgA (*r*^2^=0.23, *P* < 0.0001; Figure 1C) and IgA–IgG immune complex (*r*^2^=0.12, *P* < 0.01; Figure 1D).

### gAIM Deposition in Patients with IgA Nephropathy

**Figure 2:**
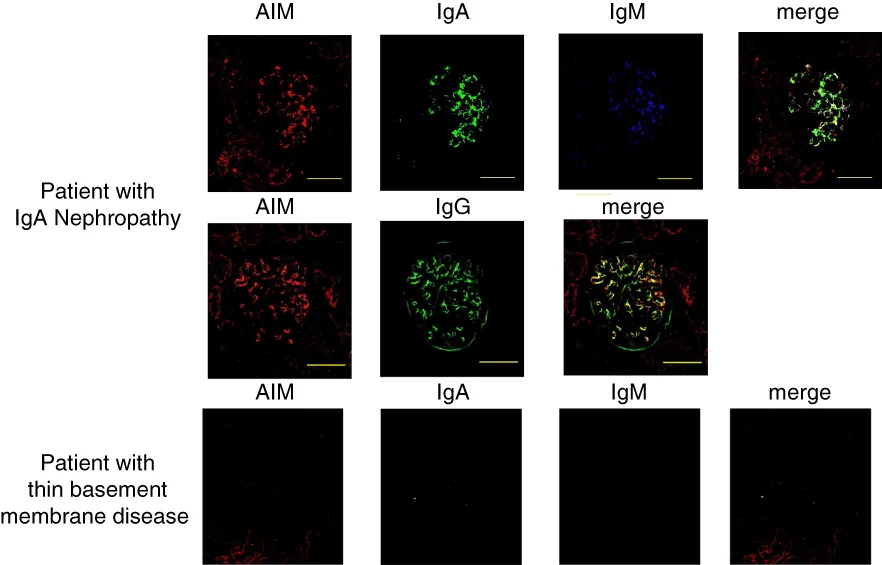
**Representative immunofluorescence staining images of mesangial AIM in patients with IgA nephropathy.** The presence of granular AIM in the mesangial and capillary areas is confirmed in patients with IgA nephropathy (above); calculated gAIM was 7.4%; immunofluorescence score of IgA deposition was 2+; IgG was 1+ and that of IgM was 1+. There was no detectable gAIM deposition in patients with thin basement membrane disease (below), and glomerular IgA, IgG, and IgM intensities were graded as 0. Scale bars: 100 *µ*m. AIM, apoptosis inhibitor of macrophage; gAIM, glomerular apoptosis inhibitor of macrophage.

**Figure 3:**
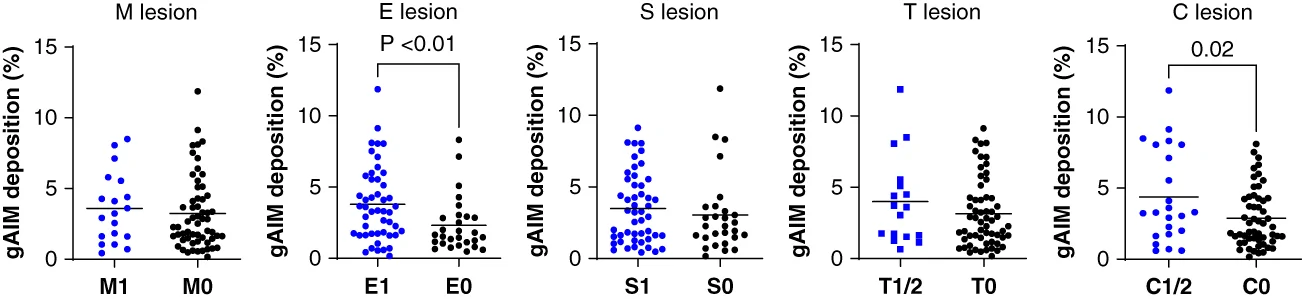
**Relationship between gAIM deposition and Oxford classification in 77 patients with IgA nephropathy.** High intensity of gAIM deposition was well correlated with acute lesions, such as Oxford E1 (E1 3% [2%–5%] versus E0 2% [1%–3%], *P* < 0.01) and C1 lesions (C1 3% [2%–8%] versus C0 2% [1%–4%], *P* < 0.05). There were no significant differences in the gAIM deposition area frequencies of Oxford M, S, and T scores. E1, endocapillary hypercellularity.

We found that gAIM deposition in the mesangial and capillary areas was colocalized with glomerular IgA in patients with IgA nephropathy, although there was no detectable gAIM deposition in patients with thin basement membrane disease (Figure 2). Compared with the low-intensity group, the high-intensity group showed significantly higher hematuria severity (30–49 versus 10–19/HPF, *P* = 0.04; Table 2). Positive correlation was confirmed between gAIM deposition with severe hematuria (*r*^2^=0.32, *P* < 0.01; Supplemental Figure 3) and proteinuria (*r*^2^=0.26, *P* = 0.02). There was no significant correlation between the sAIM levels and gAIM deposition. Other clinical manifestations showed no significant differences among the two groups. We further confirmed the relationship between gAIM deposition and pathologic manifestations. High intensity of gAIM deposition was well correlated with acute lesions, such as Oxford E1 (E1 3% [2%–5%] versus E0 2% [1%–3%], *P* < 0.01) and C1 lesions (C1 3% [2%–8%] versus C0 2% [1%–4%], *P* = 0.02). There were no significant differences in the frequencies of Oxford M, S, and T scores (Figure 3).

**Table 2:** Relationship between glomerular apoptosis inhibitor of macrophage deposition with clinical characteristics of patients with IgA nephropathy

Variable	Patients With IgA Nephropathy (*n*=77)	High-Intensity Group (≥4.0%; *n*=39)	Low-Intensity Group (<4.0%; *n*=38)
**Clinical features**			
Sex: male/female, *n*	38/39	19/20	19/19
Age, yr, mean±SD	39±15	40±18	38±13
MAP, mm Hg, mean±SD	83±9	84±10	80±7
BMI, mean±SD	23±4	23±4	23±4
**Laboratory measurements**			
Hematuria (<5, 5–9, 10–19, 30–49, ≧50/HPF), *n* (%)	5 (7), 10 (13), 16 (21), 12 (17), 34 (44)	1 (3), 4 (10), 7 (18), 6 (15), 21 (54)	4 (11), 6 (16), 9 (24), 6 (16), 13 (34)
Proteinuria, g/gCr, median (IQR)	0.6 (0.3–1.3)	0.7 (0.4–1.6)	0.5 (0.3–0.8)
Serum creatinine, mg/dl, mean±SD	0.8±0.2	0.8±0.1	0.8±0.2
eGFR, ml/min per 1.73 m^2^, mean±SD	84±18	83±18	85±18
Serum albumin, g/dl, mean±SD	4.0±0.5	4.0±0.5	4.2±0.4
Serum IgG, mg/dl, mean±SD	1041±304	963±306	1124±279
Serum IgA, mg/dl, mean±SD	307±90	306±96	308±85
Serum IgM, mg/dl, mean±SD	100±47	105±57	96±3
Serum C3, mg/dl, mean±SD	97±18	98±19	97±17
Serum Gd-IgA1, mg/dl, median (IQR)	4.5 (3.6–5.7)	4.4 (3.7–5.1)	4.6 (3.5–6.7)
Urine Gd-IgA1/UTPCr, g/gCr, median (IQR)	29.7 (17.2–66.4)	38.0 (19.6–76.1)	27.8 (16.6–44.2)
Serum IgA‑IgG immune complex, OD, median (IQR)	1.71 (1.63–1.83)	1.71 (1.61–1.83)	1.70 (1.61–1.84)
Serum IgA–IgM immune complex, OD, median (IQR)	1.78 (1.42–1.99)	1.80 (1.44–2.07)	1.76 (1.42–1.90)
Immunofluorescence, *n* (%)			
*Glomerular IgA deposition (0, 1+, 2+)*	0 (0), 0 (0), 77 (100)	0 (0), 0 (0), 39 (100)	0 (0), 0 (0), 38 (100)
*Glomerular IgG deposition (0, 1+, 2+)*	22 (29), 55 (71), 0 (0)	11 (28), 28 (72), 0 (0)	11 (29), 55 (71), 0 (0)
*Glomerular IgM deposition (0, 1+, 2+)*	19 (25), 56 (73), 2 (3)	5 (13), 32 (82),2 (5)	14 (37), 24 (63), 0 (0)
*Glomerular C3 deposition (0, 1+, 2+)*	0 (0), 20 (26), 57 (74)	0 (0),4 (10), 35 (90)	0 (0), 16 (42), 22 (58)
*Glomerular C1q deposition (0, 1+, 2+)*	77 (100), 0 (0), 0 (0)	39 (100), 0 (0), 0 (0)	38 (100), 0 (0), 0 (0)
**Treatment options, *n* (%)**			
Tonsillectomy and steroid pulse therapy	54 (70)	27 (70)	27 (71)
Steroid pulse monotherapy	3 (4)	3 (8)	0 (0)
Tonsillectomy only	14 (18)	7 (18)	7 (18)
RAS inhibitors	24 (31)	15 (38)	9 (24)

Significance was determined by the Mann–Whitney *U* test and unpaired *t* test. BMI, body mass index; C3, complement 3; gAIM, glomerular apoptosis inhibitor of macrophage; Gd-IgA1, galactose-deficient IgA1; /HPF, per high-power field; IQR, interquartile range; MAP, mean arterial BP; RAS, renin-angiotensin system.

### Association between gAIM and Glomerular Complement Component Deposition

**Figure 4:**
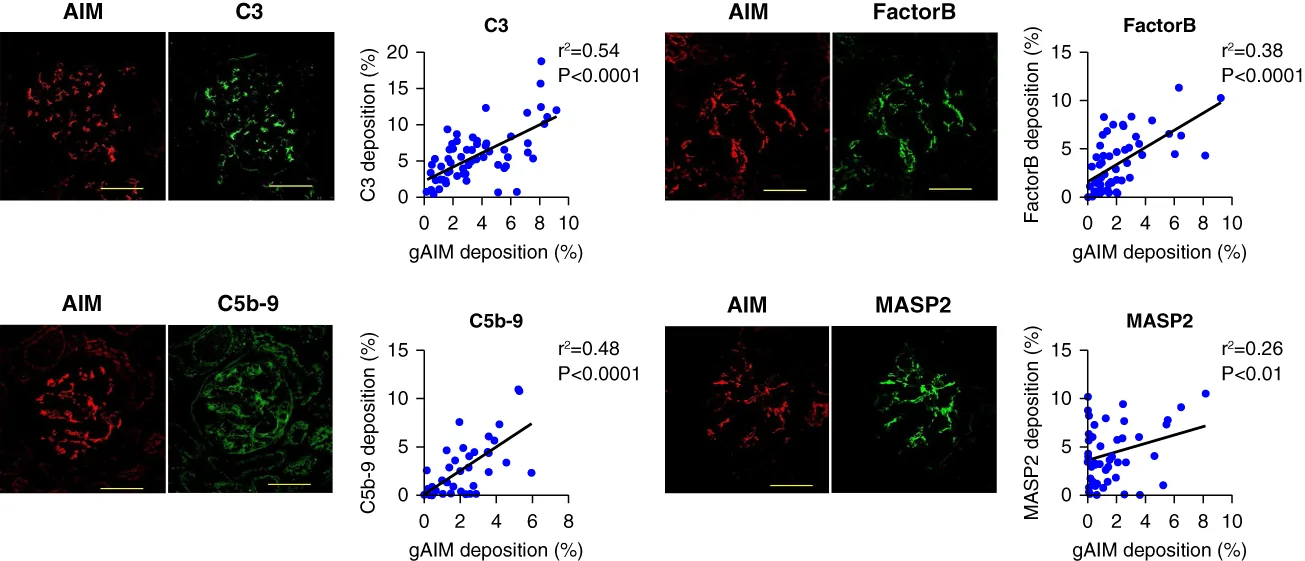
**Relationship between gAIM deposition and complement components in 77 patients with IgA nephropathy.** Representative immunostaining images of AIM (red) and C3, factor B, C5b-9, and MASP-2 (green) in the renal tissue of patients with IgA nephropathy show that gAIM deposition is positively correlated with glomerular complement component deposition. Scale bars: 100 *µ*m. C3, complement 3; MASP-2, associated serine protease-2.

We also investigated the colocalization of gAIM and complement components in patients with IgA nephropathy using immunofluorescence staining. As shown in Figure 4, gAIM deposition area had a strong positive correlation with C3 deposition (*r*^2^=0.54, *P* < 0.0001) and positive correlations with the deposition areas of the glomerular complement components factor B (*r*^2^=0.38, *P* < 0.0001), C5b-9 (*r*^2^=0.48, *P* < 0.0001), and MASP-2 (*r*^2^=0.26, *P* < 0.01).

### High sAIM Level as an Independent Predictor of Hematuria Remission in Patients with IgA Nephropathy

**Figure 5:**
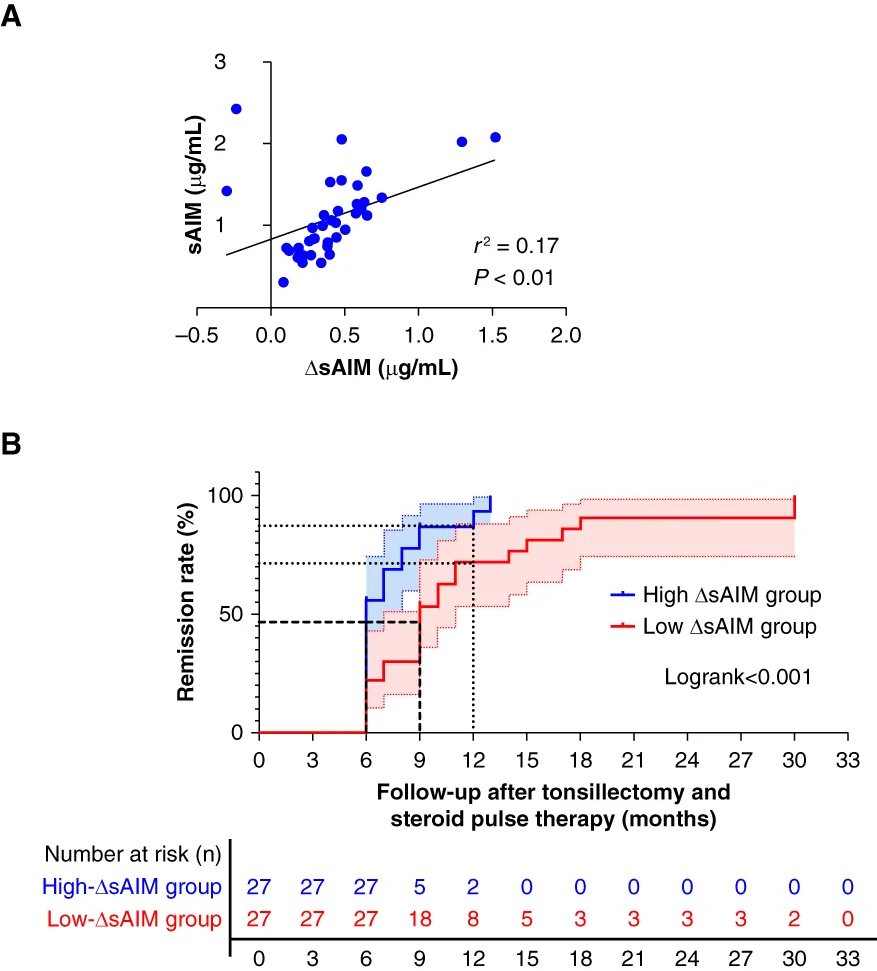
**Relationship between ΔsAIM level and hematuria remission in 54 IgA nephropathy patients with tonsillectomy and steroid pulse therapy.** (A) ΔsAIM had a positive correlation with sAIM levels at biopsy (*r*^2^=0.17, *P* < 0.01). (B) Kaplan–Meier analysis with log-rank test reveals a significant difference in the hematuria remission rate after tonsillectomy and steroid pulse therapy between the two groups divided by ΔsAIM levels (median remission time 6 versus 9 months, log-rank test; *P* < 0.001). Patients with ΔsAIM level ≥0.45 *µ*g/ml were included in the high-ΔsAIM group, whereas those with ΔsAIM level <0.45 *µ*g/ml were included in the low-ΔsAIM group. The dashed lines were 95% CIs. ΔsAIM, sAIM reduction during the therapy; CI, confidence interval.

The relationship between clinical parameters at diagnosis, such as sAIM/gAIM, and hematuria remission was assessed by univariate and multivariate Cox regression analyses among all 77 patients. Multivariate Cox regression analysis showed that high sAIM level (HR, 1.86; 95% CI, 1.03 to 3.15; *P* = 0.04) and proteinuria (HR, 1.58; 95% CI, 1.21 to 2.04; *P* < 0.01) were independent predictors of hematuria remission (Table 3). Among 54 patients who received tonsillectomy and steroid pulse therapy, we analyzed the correlation between ΔsAIM and hematuria remission. ΔsAIM had a positive correlation with sAIM levels at biopsy (*r*^2^=0.17, *P* < 0.01, Figure 5A). Using the median ΔsAIM, we classified patients into high (≥0.45 *µ*g/ml) and low (<0.45 *µ*g/ml) ΔsAIM groups. The time to reach hematuria remission from completion of the therapy was compared between the two ΔsAIM groups. No significant difference was observed in the median time interval of measuring sAIM levels between the high- and low-ΔsAIM groups (10 [9–13] versus 11 [9–14] months). However, Kaplan–Meier analysis showed that patients in the high-ΔsAIM group were associated with early remission rather than in the low-ΔsAIM group (6 versus 9 months, *P* < 0.001; Figure 5B). The median first day of negative hematuria in the high-ΔsAIM group was within 1 month after completion of the therapy. Conversely, the median time to reach proteinuria remission was not significantly different in the high-ΔsAIM group than in their respective counterparts (Supplemental Figure 4).

**Table 3:** Univariate and multivariate Cox regression analyses for predictors of hematuria remission in 77 patients with IgA nephropathy at diagnosis

Variables	Univariate Analysis	
	HR	95% CI
Sex: male/female	1.49	0.89 to 2.54
Age (continuous per 1-yr increase)	1.01	0.99 to 1.03
Hematuria (≧50/HPF)	0.92	0.80 to 1.08
Proteinuria (continuous per 1 g/gCr increase)	1.35	1.04 to 1.68
Serum creatinine (continuous per 0.1 mg/dl increase)	1.08	0.95 to 1.23
eGFR (continuous per 1 ml/min per 1.73 m^2^ decrease)	0.99	0.97 to 1.00
Serum IgA (continuous per 1 mg/dl increase)	1.00	1.00 to 1.01
Serum Gd-IgA1 (continuous per 1 mg/dl increase)	1.07	0.91 to 1.24
Serum IgA–IgG immune complex, (continuous per 0.1 OD increase)	2.67	0.72 to 8.11
Oxford E1 lesion	0.98	0.58 to 1.70
sAIM, (continuous per 1 *µ*g/ml increase)	1.90	1.12 to 3.14
gAIM deposition (continuous per 1% increase)	1.04	0.92 to 1.15

Variables	Multivariate Analysis	
	HR	95% CI
Hematuria (≧50/HPF)	0.92	0.74 to 1.13
Proteinuria	1.58	1.21 to 2.04
Oxford E1	0.72	0.34 to 1.52
sAIM	1.86	1.03 to 3.15
Serum IgA–IgG immune complex	1.96	0.47 to 6.88

Significance was determined by the Cox regression analysis. CI, confidence interval; E1, endocapillary hypercellularity; gAIM, glomerular apoptosis inhibitor of macrophage; Gd-IgA1, galactose-deficient IgA1; /HPF, per high-power field; HR, hazard ratio; sAIM, serum IgM‑free apoptosis inhibitor of macrophage.

## Discussion

In this study, we demonstrated that sAIM levels were significantly elevated at diagnosis in patients with IgA nephropathy, decreased after tonsillectomy and steroid pulse therapy, and were positively correlated with serum IgA and IgA–IgG immune complex levels. These findings suggest that sAIM reflects disease activity in human IgA nephropathy. In a previous cohort study of Japanese children with IgA nephropathy, serum AIM levels were elevated but were not significantly associated with clinical parameters,^24^ indicating that the clinical significance of circulating AIM may differ according to disease stage, age, or disease activity.

AIM is known to circulate predominantly in an IgM-bound form, while dissociation into IgM-free AIM occurs as a sensitive physiologic response to pathologic conditions and has been proposed as a disease biomarker in various inflammatory diseases.^15,23,25–27^ In the context of IgA nephropathy, one plausible explanation is that nephritogenic IgA or IgA-containing immune complexes trigger the release of AIM from pentameric IgM, resulting in elevated sAIM levels that reflect ongoing immune complex–mediated glomerular injury. Alternatively, qualitative or structural abnormalities of pathogenic IgA may facilitate its interaction with AIM. Our previous study demonstrated that AIM associates with IgA in both mice and humans and binds to the IgA Fc region in mice.^11^ However, whether circulating sAIM directly contributes to *in situ* immune complex formation in human glomeruli remains unclear. Moreover, because AIM does not bind IgG, its involvement in IgA–IgG immune complex formation is likely indirect and requires further mechanistic investigation.

A previous study reported the presence of AIM in various kidney diseases and that codeposition of AIM and IgM was associated with severe proteinuria and advanced kidney dysfunction.^28^ However, the disease specificity of AIM has not been previously reported. In this study, we found gAIM deposition in the mesangial and capillary lesions with glomerular IgA in patients with IgA nephropathy. The high-intensity gAIM deposition group showed more severe hematuria than the low-intensity group and had relatively more frequent pathologic manifestations of acute lesions (*i.e*., Oxford E1 and C1). Moreover, high gAIM deposition intensity had a strong correlation with glomerular complement activation. Notably, the deposition of immune complexes induces complement activation in the pathogenesis of IgA nephropathy; however, this study showed an abnormally strong correlation between gAIM deposition and complement deposition. Maehara *et al.* reported that accumulated AIM on the cancer cell surface caused *in situ* complement activation and played a beneficial role in preventing hepatocellular carcinoma proliferation,^29^ suggesting that accumulated AIM may functionally suppress multiple complement-regulating factors. Recent findings suggested that the imbalance between factor H-related protein 5 and factor H, which mainly regulate alternative pathway activation, may increase the severity of IgA nephropathy by accentuating glomerular injury through complement activation.^30,31^ Although the complete mechanism underlying gAIM-mediated complement activation in IgA nephropathy pathogenesis remains to be elucidated, AIM-mediated complement activation is likely involved in glomerular inflammation. Because gAIM accurately reflects the acute glomerular injuries by complement activation, gAIM may be an important marker for selecting complement-targeting agents for patients with IgA nephropathy.

Whether gAIM comes from circulating AIM (IgM-bound AIM and/or sAIM) and/or *in situ* AIM accumulation from tissue macrophages remains controversial. If gAIM originates from circulating AIM, sAIM levels should be expected to be correlated with gAIM deposition. In our cohort, there was no significant correlation of sAIM with gAIM, and high gAIM intensity and high sAIM levels did not exhibit similar clinical characteristics. Recently, it is demonstrated that the IgA–IgM immune complex has a strong effect on complement activation inducing local inflammation. Matsumoto *et al.* demonstrated that anti‑Gd-IgA1 IgM was present and formed immune complex in human IgA nephropathy serum samples and that this immune complex contained abundant C3.^32^ Actually, glomerular inflammation using AIM may be caused by mainly IgA–IgM immune complexes containing AIM bound with pentamer IgM or IgA, which are produced according to disease activity. These data may provide a possible explanation for our results that sAIM and gAIM had no significant association. Another possibility is *in situ* immune complex formation on glomeruli by AIM from tissue macrophages. The complete picture of circulating immune complex with gAIM in IgA nephropathy pathogenesis requires further evaluation.

Hematuria is one of the most common manifestations in patients with IgA nephropathy. In recent studies, persistent hematuria over time was found to significantly influence progression to ESKD, and improvement of hematuria significantly decreased the rate of renal function loss.^33,34^ In a Spanish cohort of 112 patients with IgA nephropathy, Sevillano *et al.* assessed the association of longitudinal change in microscopic hematuria with subsequent kidney events. It was observed that patients with consistently elevated consistent hematuria had a significantly larger decrease in renal function when compared with their counterparts who had negligible or no consistent.^33^ Yano *et al.* assessed the association between urine dipsticks hematuria and proteinuria trajectory patterns among patients with IgA nephropathy and risk of events of a ≥50% eGFR decline and mean annual eGFR decline rate. They observed that patients with persistent hematuria during follow-up, regardless of the trajectory patterns of proteinuria, are at higher risk of kidney events.^34^ We previously found that the disappearance or improvement of hematuria after tonsillectomy and steroid pulse therapy was correlated with decreased serum IgA–IgG immune complex levels and demonstrated that serum IgA–IgG immune complex level could serve as a useful marker in human IgA nephropathy.^7^ In this study, we found that high-sAIM level was an independent predictor of hematuria remission and ΔsAIM level reflected hematuria remission. These results suggest that the sAIM reflects disease activity and prognosis of hematuria remission in human IgA nephropathy.

Several previous observational retrospective studies identified the degree of proteinuria as one of the most important risk factors of ESKD,^35^ and its reduction has been accepted as a surrogate end point in IgA nephropathy.^36,37^ In this study, sAIM and gAIM were not associated with proteinuria degree at diagnosis and during the disease course. One possible reason for this result could be that proteinuria in IgA nephropathy can be caused by both acute and chronic lesions. The chronic lesions defined as the presence of S1 lesion is widely recognized to have a correlation with a relatively high degree of proteinuria at diagnosis and is associated with rapid loss of kidney function and worse kidney survival.^22^ S1 lesions in IgA nephropathy are believed to be caused by postinflammatory scarring, compensatory hemodynamic changes after nephron loss, and primary podocyte damage secondary to mediators from mesangial cells.^38^ In this study, we excluded patients with renal failure and 22% of patients had T1 lesion. Considering that most of our cohort suggested being in the early stage of the clinical course in IgA nephropathy, our data may be insufficient to show that gAIM and sAIM are involved in the chronic lesion formation, such as S1 lesion, associated with sustained proteinuria.

However, we should not overlook the possibility of AIM involvement in renal fibrosis in patients with IgA nephropathy. Yang *et al.* found AIM expression in renal tubular epithelial cells and lumen, and its expression area in renal tissues had correlations with the fibrotic area, 24-hour urine protein, and serum creatinine in patients with IgA nephropathy. They also demonstrated the possible involvement of AIM in renal fibrosis through a mechanism mediated by TGF *β*1 and M2 macrophages.^39^ Together with the results of this study, AIM was suggested to be involved in acute lesion formation and chronic renal fibrosis by different mechanisms. To use AIM as a biomarker for human IgA nephropathy, the prospective validation about the long-term influence of glomerular and circulating AIM on the clinical course is necessary.

This study had some limitations that need to be considered. First, this was a retrospective study with a small population that included only Japanese patients with IgA nephropathy; inclusion of more diverse ethnic groups from multiple centers is required. Second, exclusion of patients with hypertension, hyperlipidemia, diabetes, or any other conditions that may be influenced by AIM might have been insufficient. Finally, this study only observed the short-term clinical course until remission of urinary findings. Long-term follow-up data are required to analyze the relationship between AIM and renal prognosis.

In conclusion, our results suggest that gAIM was associated with complement activation and involved in glomerular inflammation with acute lesions and hematuria. Moreover, sAIM had a positive correlation with the serum level of IgA–IgG immune complex and reflected hematuria remission after tonsillectomy and steroid pulse therapy. We believe that sAIM and gAIM could serve as one of the useful biomarkers of disease activity and prognosis of hematuria in human IgA nephropathy.

## Supplementary Material

## Data Availability

Original data generated for the study will be made available upon reasonable request to the corresponding author. Data Type: Raw Data/Source Data, Observational Data, Research Protocols, Survey Data, and Statistical Analysis Plan. Reason for Restricted Access: Regarding human data, the data are not publicly available due to ethical restrictions approved by the ethics committee of Juntendo University Hospital. Data will be available on the reasonable request.

## Declarative Statements

This study includes clinical experimentation and received Institutional Review Board or Ethics Committee approval. The need to obtain informed patient consent was waived. This study includes clinical experimentation and complies with the Declaration of Helsinki.

## Supplemental Material

Supplemental Figure 1. Flow diagram of the study design.

Supplemental Figure 2. Treatment and follow-up design.

Supplemental Figure 3. Relationship between gAIM deposition and clinical variables in 77 patients with IgA nephropathy.

Supplemental Figure 4. Kaplan–Meier survival curves of the time to proteinuria remission event in 54 IgA nephropathy patients with tonsillectomy and steroid pulse therapy.
