# Supplementary material for: Apoptosis Inhibitor of Macrophage and Acute Inflammation in IgA Nephropathy
Source: Kidney360. 2026 May 22;7(8):1842–53. doi: 10.34067/KID.0000001202 (PMC13567846; doi:10.34067/KID.0000001202)
Supplement: Supplementary file 1 [file kidney360-7-1842-s001.pdf]

Supplemental Material Table of Contents

This article contains the following supplemental materials:

- Supplemental Figure 1. Flow diagram of the study design
- Supplemental Figure 2. Treatment and follow-up design
- Supplemental Figure 3. Relationship between glomerular AIM (gAIM) deposition and clinical variables in 77 patients with IgA nephropathy
- Supplemental Figure 4. The Kaplan–Meier survival curves of time to proteinuria remission event in 54 IgA nephropathy patients

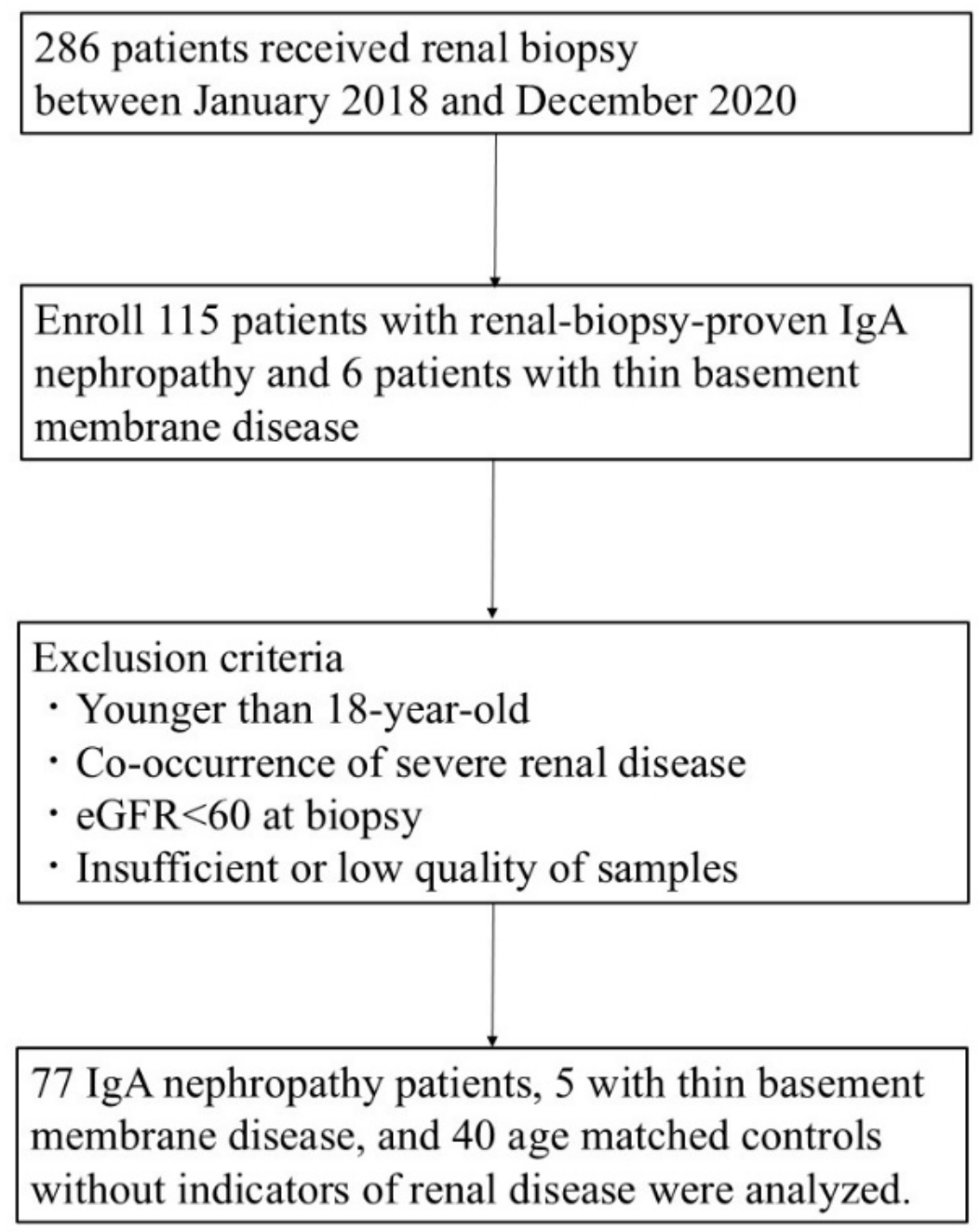

Supplemental Figure 1. Flow diagram of the study design

This study enrolled 77 IgA nephropathy patients, 5 thin basement membrane disease patients, and 40 healthy controls.

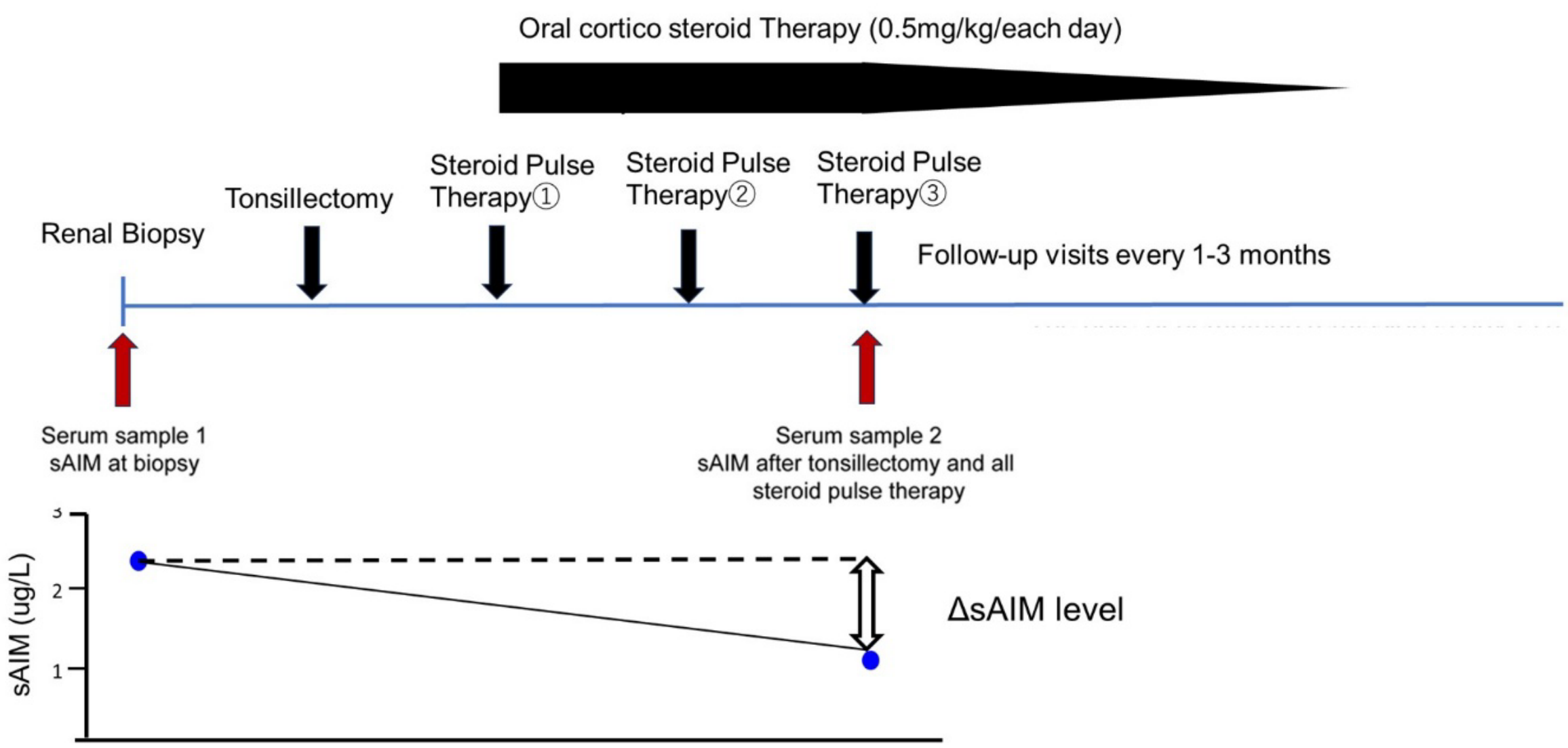

- **Supplemental Figure 2. Treatment and follow-up design**

Steroid pulse therapy (intravenous 500 mg methylprednisolone per day for 3 consecutive days, thrice every 2 months) was administered after tonsillectomy. Between sessions of steroid pulse therapy, patients were prescribed oral prednisolone (0.5 mg/kg body weight) on alternate days. All patients measured sAIM levels at biopsy and on the last day of steroid pulse therapy and were calculated for ΔsAIM level. Patients were scheduled for regular follow-up visits at intervals of 1–3 months, and the dose of oral prednisolone was gradually tapered off. Urine sediments and proteinuria were tested at every visit conducted at 1 to 3 months throughout the follow-up period. Hematuria remission was defined as three consecutive red blood cell count of <5 per high-power field for at least 6 months

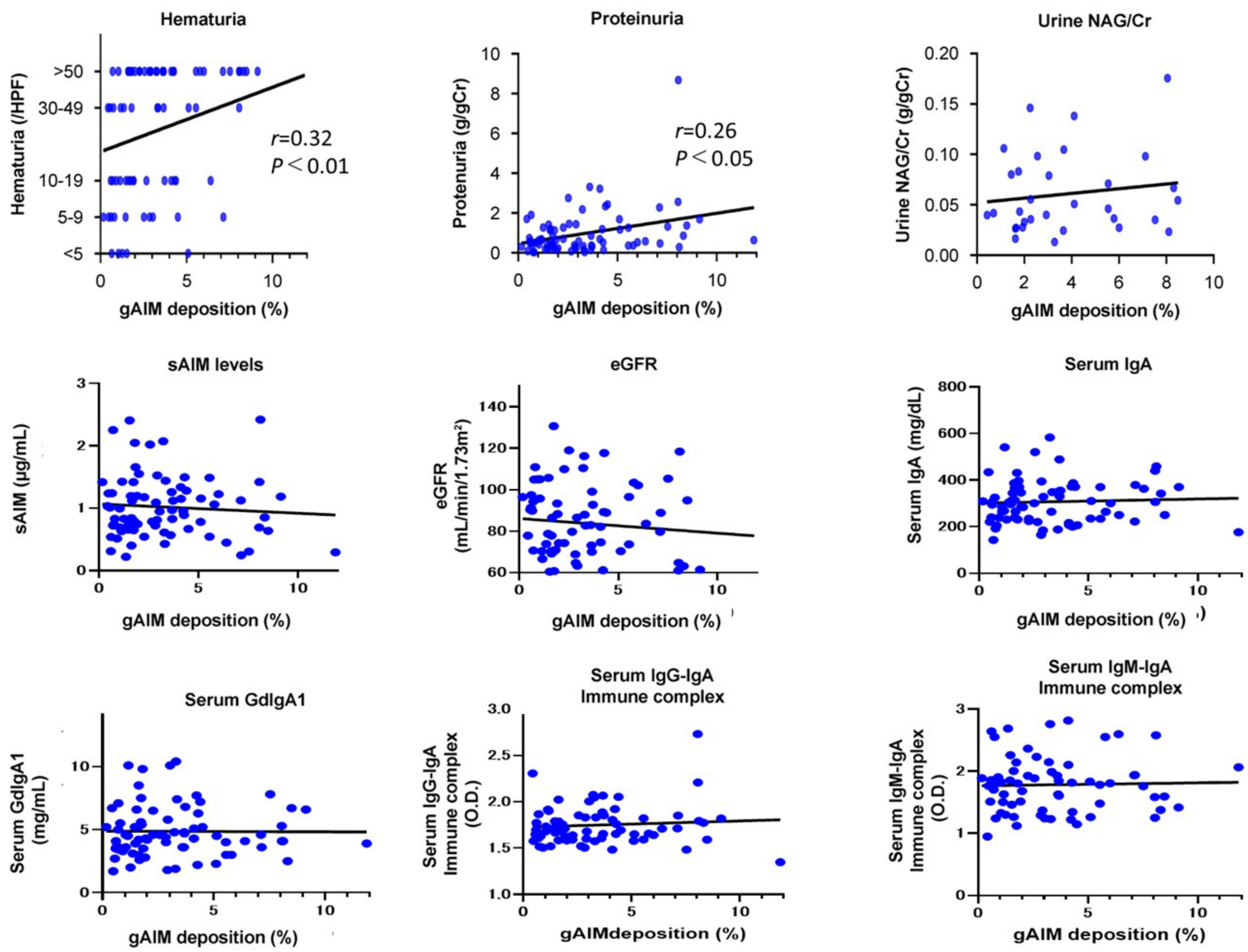

Supplemental Figure 3. Relationship between glomerular AIM (gAIM) deposition and clinical variables in 77 patients with IgA nephropathy

(A) The gAIM deposition was positively correlated with the degree of hematuria ($r = 0.32$, $P < 0.01$) and proteinuria ($r = 0.26$, $P = 0.02$). Results were calculated using Pearson and Spearman correlation coefficients and have been presented in the graph.

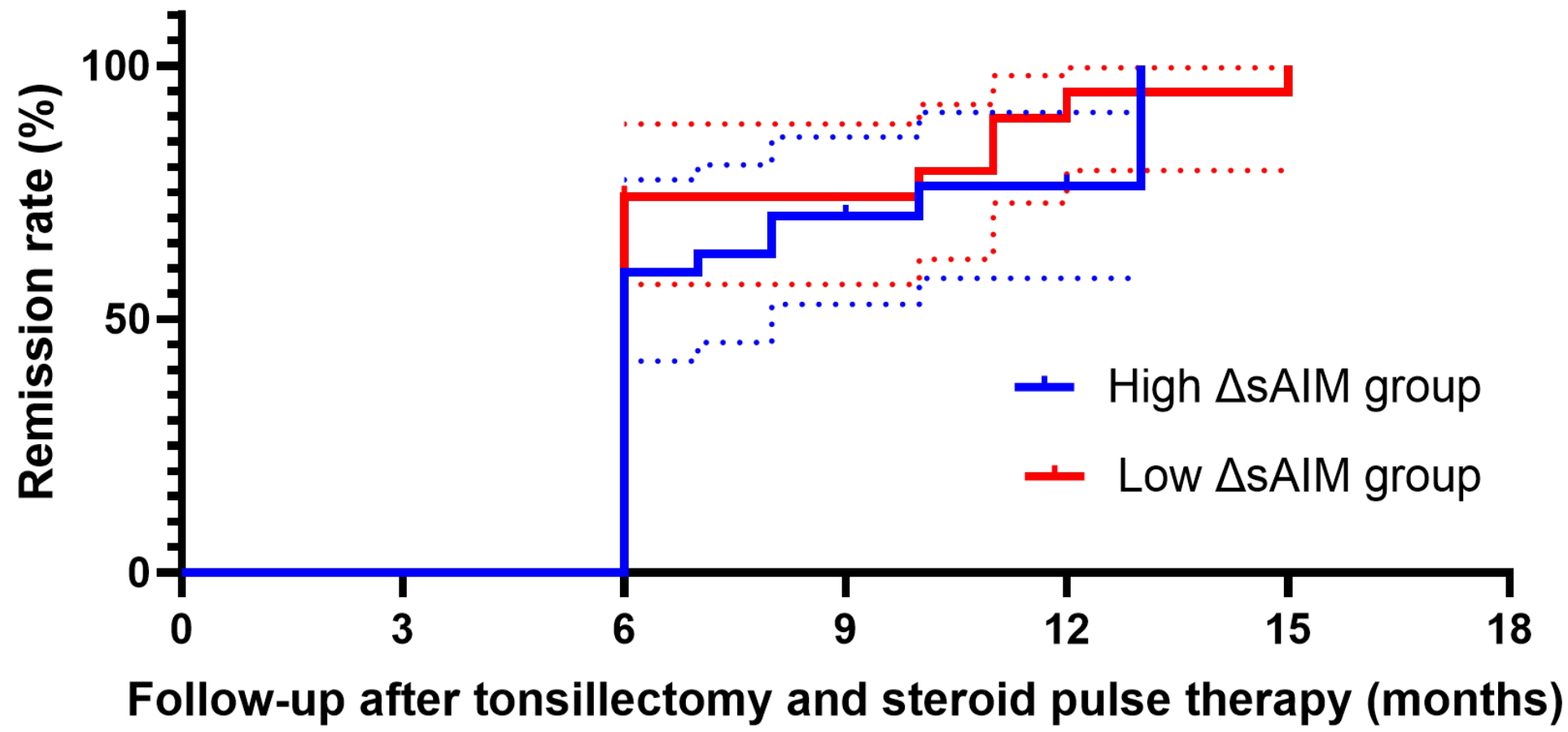

Number at risk (n)							
High-ΔsAIM group	27	27	27	8	4	0	0
Low-ΔsAIM group	27	27	27	27	2	1	0
	0	3	6	9	12	15	18

Supplemental Figure 4. The Kaplan–Meier survival curves of time to proteinuria remission event in 54 IgA nephropathy patients

Kaplan–Meier analysis with log-rank test reveals no significant difference in the proteinuria remission rate after tonsillectomy and steroid pulse therapy among the two groups divided by ΔsAIM levels. Patients with ΔsAIM level ≥0.45 μg/mL were included in the high ΔsAIM group (blue), whereas those with ΔsAIM level <0.45 μg/mL were included in the low ΔsAIM group (red). The dashed lines were 95% Confidence Intervals.
