# Supplementary material for: Apoptosis Inhibitor of Macrophage and Acute Inflammation in IgA Nephropathy
Source: Kidney360. 2026 May 22;7(8):1842–53. doi: 10.34067/KID.0000001202 (PMC13567846; doi:10.34067/KID.0000001202)
Supplement: Supplementary file 2 [file kidney360-7-1842-s002.pdf]

ASN American Society of Nephrology | JASN Journal of the American Society of Nephrology | KIDNEY360 Accessing Our World From Every Angle | CJASN Clinical Journal of the American Society of Nephrology

# ASN Journal Disclosure Form

As per ASN journal policy, I have disclosed any financial relationships or commitments I have held in the past 36 months as included below. I have listed my Current Employer below to indicate there is a relationship requiring disclosure. If no relationship exists, my Current Employer is not listed.

R. Aoki reports the following:
Employer: Juntendo University

I understand that the information above will be published within the journal article, if accepted, and that failure to comply and/or to accurately and completely report the potential financial conflicts of interest could lead to the following: 1) Prior to publication, article rejection, or 2) Post-publication, sanctions ranging from, but not limited to, issuing a correction, reporting the inaccurate information to the authors' institution, banning authors from submitting work to ASN journals for varying lengths of time, and/or retraction of the published work.

Name: Ryousuke Aoki
Manuscript ID: JASN-2025-000606R1
Manuscript Title: Apoptosis inhibitor of macrophage and acute inflammation in IgA nephropathy
Date of Completion: September 13, 2025
Disclosure Updated Date: September 13, 2025

ASN American Society of Nephrology
JASN Journal of the American Society of Nephrology
KIDNEY360 Accessing Our World From Every Angle
CJASN Clinical Journal of the American Society of Nephrology

# ASN Journal Disclosure Form

As per ASN journal policy, I have disclosed any financial relationships or commitments I have held in the past 36 months as included below. I have listed my Current Employer below to indicate there is a relationship requiring disclosure. If no relationship exists, my Current Employer is not listed.

S. Arai reports the following:
Ownership Interest: IAM CAT, Inc.

I understand that the information above will be published within the journal article, if accepted, and that failure to comply and/or to accurately and completely report the potential financial conflicts of interest could lead to the following: 1) Prior to publication, article rejection, or 2) Post-publication, sanctions ranging from, but not limited to, issuing a correction, reporting the inaccurate information to the authors' institution, banning authors from submitting work to ASN journals for varying lengths of time, and/or retraction of the published work.

Name: Satoko Arai
Manuscript ID: JASN-2025-000606R1
Manuscript Title: Apoptosis inhibitor of macrophage and acute inflammation in IgA nephropathy
Date of Completion: September 13, 2025
Disclosure Updated Date: September 13, 2025

ASN American Society of Nephrology · JASN Journal of the American Society of Nephrology · KIDNEY360 Accessing Our World From Every Angle · CJASN Clinical Journal of the American Society of Nephrology

# ASN Journal Disclosure Form

As per ASN journal policy, I have disclosed any financial relationships or commitments I have held in the past 36 months as included below. I have listed my Current Employer below to indicate there is a relationship requiring disclosure. If no relationship exists, my Current Employer is not listed.

Y. Fukao reports the following:
Employer: Juntendo University Faculty of Medicine

I understand that the information above will be published within the journal article, if accepted, and that failure to comply and/or to accurately and completely report the potential financial conflicts of interest could lead to the following: 1) Prior to publication, article rejection, or 2) Post-publication, sanctions ranging from, but not limited to, issuing a correction, reporting the inaccurate information to the authors' institution, banning authors from submitting work to ASN journals for varying lengths of time, and/or retraction of the published work.

Name: Yusuke Fukao
Manuscript ID: JASN-2025-000606R1
Manuscript Title: Apoptosis inhibitor of macrophage and acute inflammation in IgA nephropathy
Date of Completion: September 6, 2025
Disclosure Updated Date: September 6, 2025

ASN American Society of Nephrology | JASN Journal of the American Society of Nephrology | KIDNEY360 Accessing Our World From Every Angle | CJASN Clinical Journal of the American Society of Nephrology

# ASN Journal Disclosure Form

As per ASN journal policy, I have disclosed any financial relationships or commitments I have held in the past 36 months as included below. I have listed my Current Employer below to indicate there is a relationship requiring disclosure. If no relationship exists, my Current Employer is not listed.

T. Kano reports the following:
Employer: Juntendo University Faculty of Medicine

I understand that the information above will be published within the journal article, if accepted, and that failure to comply and/or to accurately and completely report the potential financial conflicts of interest could lead to the following: 1) Prior to publication, article rejection, or 2) Post-publication, sanctions ranging from, but not limited to, issuing a correction, reporting the inaccurate information to the authors' institution, banning authors from submitting work to ASN journals for varying lengths of time, and/or retraction of the published work.

Name: Toshiki Kano
Manuscript ID: K360-2025-001169R4
Manuscript Title: Apoptosis inhibitor of macrophage and acute inflammation in IgA nephropathy
Date of Completion: March 26, 2026
Disclosure Updated Date: December 23, 2025

# ASN Journal Disclosure Form

As per ASN journal policy, I have disclosed any financial relationships or commitments I have held in the past 36 months as included below. I have listed my Current Employer below to indicate there is a relationship requiring disclosure. If no relationship exists, my Current Employer is not listed.

R. Kato has nothing to disclose.

I understand that the information above will be published within the journal article, if accepted, and that failure to comply and/or to accurately and completely report the potential financial conflicts of interest could lead to the following: 1) Prior to publication, article rejection, or 2) Post-publication, sanctions ranging from, but not limited to, issuing a correction, reporting the inaccurate information to the authors' institution, banning authors from submitting work to ASN journals for varying lengths of time, and/or retraction of the published work.

Name: Rina Kato
Manuscript ID: JASN-2025-000606R1
Manuscript Title: Apoptosis inhibitor of macrophage and acute inflammation in IgA nephropathy
Date of Completion: September 5, 2025
Disclosure Updated Date: September 5, 2025

ASN American Society of Nephrology | JASN Journal of the American Society of Nephrology | KIDNEY360 Accessing Our World From Every Angle | CJASN Clinical Journal of the American Society of Nephrology

# ASN Journal Disclosure Form

As per ASN journal policy, I have disclosed any financial relationships or commitments I have held in the past 36 months as included below. I have listed my Current Employer below to indicate there is a relationship requiring disclosure. If no relationship exists, my Current Employer is not listed.

A. Koizumi reports the following:
Employer: Juntendo University Faculty of Medicine; and Research Funding: argenx BV.

I understand that the information above will be published within the journal article, if accepted, and that failure to comply and/or to accurately and completely report the potential financial conflicts of interest could lead to the following: 1) Prior to publication, article rejection, or 2) Post-publication, sanctions ranging from, but not limited to, issuing a correction, reporting the inaccurate information to the authors' institution, banning authors from submitting work to ASN journals for varying lengths of time, and/or retraction of the published work.

Name: Ayako Koizumi
Manuscript ID: K360-2025-001169R2
Manuscript Title: Apoptosis inhibitor of macrophage and acute inflammation in IgA nephropathy
Date of Completion: March 20, 2026
Disclosure Updated Date: March 20, 2026

ASN American Society of Nephrology · JASN Journal of the American Society of Nephrology · KIDNEY360 Accessing Our World From Every Angle · CJASN Clinical Journal of the American Society of Nephrology

# ASN Journal Disclosure Form

As per ASN journal policy, I have disclosed any financial relationships or commitments I have held in the past 36 months as included below. I have listed my Current Employer below to indicate there is a relationship requiring disclosure. If no relationship exists, my Current Employer is not listed.

Y. Makita reports the following:
Employer: Juntendo Universty Faculty of Medicine

I understand that the information above will be published within the journal article, if accepted, and that failure to comply and/or to accurately and completely report the potential financial conflicts of interest could lead to the following: 1) Prior to publication, article rejection, or 2) Post-publication, sanctions ranging from, but not limited to, issuing a correction, reporting the inaccurate information to the authors' institution, banning authors from submitting work to ASN journals for varying lengths of time, and/or retraction of the published work.

Name: Yuko Makita
Manuscript ID: K360-2025-001169R1
Manuscript Title: Apoptosis inhibitor of macrophage and acute inflammation in IgA nephropathy,
Date of Completion: December 23, 2025
Disclosure Updated Date: December 23, 2025

ASN American Society of Nephrology | JASN Journal of the American Society of Nephrology | KIDNEY360 Accessing Our World From Every Angle | CJASN Clinical Journal of the American Society of Nephrology

# ASN Journal Disclosure Form

As per ASN journal policy, I have disclosed any financial relationships or commitments I have held in the past 36 months as included below. I have listed my Current Employer below to indicate there is a relationship requiring disclosure. If no relationship exists, my Current Employer is not listed.

T. Miyazaki reports the following:
Employer: The Institute for AIM Medicine

I understand that the information above will be published within the journal article, if accepted, and that failure to comply and/or to accurately and completely report the potential financial conflicts of interest could lead to the following: 1) Prior to publication, article rejection, or 2) Post-publication, sanctions ranging from, but not limited to, issuing a correction, reporting the inaccurate information to the authors' institution, banning authors from submitting work to ASN journals for varying lengths of time, and/or retraction of the published work.

Name: Toru Miyazaki
Manuscript ID: JASN-2025-000606R1
Manuscript Title: Apoptosis inhibitor of macrophage and acute inflammation in IgA nephropathy
Date of Completion: September 12, 2025
Disclosure Updated Date: September 12, 2025

ASN American Society of Nephrology JASN Journal of the American Society of Nephrology KIDNEY360 Accessing Our World From Every Angle CJASN Clinical Journal of the American Society of Nephrology

# ASN Journal Disclosure Form

As per ASN journal policy, I have disclosed any financial relationships or commitments I have held in the past 36 months as included below. I have listed my Current Employer below to indicate there is a relationship requiring disclosure. If no relationship exists, my Current Employer is not listed.

M. Muto reports the following:
Employer: Juntendo university faculty of medicine

I understand that the information above will be published within the journal article, if accepted, and that failure to comply and/or to accurately and completely report the potential financial conflicts of interest could lead to the following: 1) Prior to publication, article rejection, or 2) Post-publication, sanctions ranging from, but not limited to, issuing a correction, reporting the inaccurate information to the authors' institution, banning authors from submitting work to ASN journals for varying lengths of time, and/or retraction of the published work.

Name: Masahiro Muto
Manuscript ID: JASN-2025-000606R1
Manuscript Title: Apoptosis inhibitor of macrophage and acute inflammation in IgA nephropathy
Date of Completion: September 5, 2025
Disclosure Updated Date: September 5, 2025

ASN American Society of Nephrology · JASN Journal of the American Society of Nephrology · KIDNEY360 Accessing Our World From Every Angle · CJASN Clinical Journal of the American Society of Nephrology

# ASN Journal Disclosure Form

As per ASN journal policy, I have disclosed any financial relationships or commitments I have held in the past 36 months as included below. I have listed my Current Employer below to indicate there is a relationship requiring disclosure. If no relationship exists, my Current Employer is not listed.

M. Nakayama reports the following:
Employer: Department of Nephrology, Juntendo University Faculty of Medicine

I understand that the information above will be published within the journal article, if accepted, and that failure to comply and/or to accurately and completely report the potential financial conflicts of interest could lead to the following: 1) Prior to publication, article rejection, or 2) Post-publication, sanctions ranging from, but not limited to, issuing a correction, reporting the inaccurate information to the authors' institution, banning authors from submitting work to ASN journals for varying lengths of time, and/or retraction of the published work.

Name: Maiko Nakayama
Manuscript ID: JASN-2025-000606R1
Manuscript Title: Apoptosis inhibitor of macrophage and acute inflammation in IgA nephropathy
Date of Completion: September 5, 2025
Disclosure Updated Date: September 5, 2025

ASN American Society of Nephrology
JASN Journal of the American Society of Nephrology
KIDNEY360 Accessing Our World From Every Angle
CJASN Clinical Journal of the American Society of Nephrology

# ASN Journal Disclosure Form

As per ASN journal policy, I have disclosed any financial relationships or commitments I have held in the past 36 months as included below. I have listed my Current Employer below to indicate there is a relationship requiring disclosure. If no relationship exists, my Current Employer is not listed.

Y. Nihei reports the following:
Honoraria: Otsuka Pharmaceutical, Novartis, Boehringer Ingelheim

I understand that the information above will be published within the journal article, if accepted, and that failure to comply and/or to accurately and completely report the potential financial conflicts of interest could lead to the following: 1) Prior to publication, article rejection, or 2) Post-publication, sanctions ranging from, but not limited to, issuing a correction, reporting the inaccurate information to the authors' institution, banning authors from submitting work to ASN journals for varying lengths of time, and/or retraction of the published work.

Name: Yoshihito Nihei
Manuscript ID: K360-2025-001169R1
Manuscript Title: Apoptosis inhibitor of macrophage and acute inflammation in IgA nephropathy,
Date of Completion: December 23, 2025
Disclosure Updated Date: September 6, 2025

ASN American Society of Nephrology · JASN Journal of the American Society of Nephrology · KIDNEY360 Accessing Our World From Every Angle · CJASN Clinical Journal of the American Society of Nephrology

# ASN Journal Disclosure Form

As per ASN journal policy, I have disclosed any financial relationships or commitments I have held in the past 36 months as included below. I have listed my Current Employer below to indicate there is a relationship requiring disclosure. If no relationship exists, my Current Employer is not listed.

H. Suzuki reports the following:
Employer: Juntendo University Urayasu Hospital; and Advisory or Leadership Role: Otsuka Pharmaceutical Advisory board member.

I understand that the information above will be published within the journal article, if accepted, and that failure to comply and/or to accurately and completely report the potential financial conflicts of interest could lead to the following: 1) Prior to publication, article rejection, or 2) Post-publication, sanctions ranging from, but not limited to, issuing a correction, reporting the inaccurate information to the authors' institution, banning authors from submitting work to ASN journals for varying lengths of time, and/or retraction of the published work.

Name: Hitoshi Suzuki
Manuscript ID: K360-2025-001169R3
Manuscript Title: Apoptosis inhibitor of macrophage and acute inflammation in IgA nephropathy
Date of Completion: March 17, 2026
Disclosure Updated Date: September 4, 2025

ASN
American Society of Nephrology
JASN
Journal of the American Society of Nephrology
KIDNEY360
Accessing Our World From Every Angle
CJASN
Clinical Journal of the American Society of Nephrology

# ASN Journal Disclosure Form

As per ASN journal policy, I have disclosed any financial relationships or commitments I have held in the past 36 months as included below. I have listed my Current Employer below to indicate there is a relationship requiring disclosure. If no relationship exists, my Current Employer is not listed.

Y. Suzuki reports the following:
Employer: Juntendo University Facultyl of Medicine; Consultancy: Otsuka America Pharmaceutical, Inc. / Chinook Therapeutics U.S., Inc. / Novartis Pharma K.K./ ARGENX IIP BV /; Alexion Pharmaceuticals, Inc. / Renalys Japan /Alpine Immune Sciences, Inc. / BioCryst Pharmaceuticals, Inc./; Takeda Pharmaceutical, Inc./Hi-Bio, Inc;; Research Funding: Sumitomo Pharma Co., Ltd. /Baxter Limited / Chugai ; Pharmaceutical Co., Ltd. / Astellas Pharma Inc. / Takeda Pharmaceutical; Company Limited / Kyowa Kirin Co, Ltd. / ; TEIJIN PHARMA LIMITED. / DAIICHI SANKYO COMPANY, LIMITED / SANWA KAGAKU KENKYUSHO CO., LTD. / ; ONO PHARMACEUTICAL CO., LTD. / Torii Pharmaceutical Co.,Ltd /Astra Zeneka/Tanabe Mitsubishi/Eisai/Boeringer/Kissei/Mochida/Bayer/Visterra&Otsuka US/ Retrophin-Travere pharmaceutical/Moderna/Chinook/Pfizer/Argenx; Honoraria: Tanabe Mitsubishi Pharmaceutical company/Kyowa Kirin/Novartis/Astellas/Takeda/Daiichi Sankyo/Sumitomo Dainippon/Astra Zeneka/Bayer/; and Advisory or Leadership Role: Novartis Pharma Co. Ltd/Chinook Therapeutics, Visterra/Otsuka Inc, Travere/Retrophin Inc, Morphosys Inc., ARGENX Inc., Kyowa Kirin Co., Ltd, ;.

I understand that the information above will be published within the journal article, if accepted, and that failure to comply and/or to accurately and completely report the potential financial conflicts of interest could lead to the following: 1) Prior to publication, article rejection, or 2) Post-publication, sanctions ranging from, but not limited to, issuing a correction, reporting the inaccurate information to the authors' institution, banning authors from submitting work to ASN journals for varying lengths of time, and/or retraction of the published work.

Name: Yusuke Suzuki
Manuscript ID: K360-2025-001169R6
Manuscript Title: Apoptosis inhibitor of macrophage and acute inflammation in IgA nephropathy.
Date of Completion: April 9, 2026
Disclosure Updated Date: April 3, 2026

ASN American Society of Nephrology | JASN Journal of the American Society of Nephrology | KIDNEY360 Accessing Our World From Every Angle | CJASN Clinical Journal of the American Society of Nephrology

# ASN Journal Disclosure Form

As per ASN journal policy, I have disclosed any financial relationships or commitments I have held in the past 36 months as included below. I have listed my Current Employer below to indicate there is a relationship requiring disclosure. If no relationship exists, my Current Employer is not listed.

K. Yasuda reports the following:
Employer: The Institute for AIM Medicine; Research Funding: The Institute for AIM Medicine; and Advisory or Leadership Role: Rimi Corporation.

I understand that the information above will be published within the journal article, if accepted, and that failure to comply and/or to accurately and completely report the potential financial conflicts of interest could lead to the following: 1) Prior to publication, article rejection, or 2) Post-publication, sanctions ranging from, but not limited to, issuing a correction, reporting the inaccurate information to the authors' institution, banning authors from submitting work to ASN journals for varying lengths of time, and/or retraction of the published work.

Name: Keisuke Yasuda
Manuscript ID: K360-2025-001169R5
Manuscript Title: Apoptosis inhibitor of macrophage and acute inflammation in IgA nephropathy
Date of Completion: April 5, 2026
Disclosure Updated Date: March 27, 2026
